# DNA barcoding of fogged caterpillars in Peru: A novel approach for unveiling host-plant relationships of tropical moths (Insecta, Lepidoptera)

**DOI:** 10.1371/journal.pone.0224188

**Published:** 2020-01-30

**Authors:** Axel Hausmann, Juliane Diller, Jerome Moriniere, Amelie Höcherl, Andreas Floren, Gerhard Haszprunar

**Affiliations:** 1 SNSB-Zoologische Staatssammlung München, München, Germany; 2 Advanced Identification Methods GmbH, München, Germany; 3 Julius Maximilians Universität, Würzburg, Germany; Tierarztliche Hochschule Hannover, GERMANY

## Abstract

The present study aimed to perform molecular identification of lepidopteran larvae from canopy fogging including gut-content analyses. A total of 130 lepidopteran larvae were selected from 37 fogging samples at the Panguana station, district Yuyapichis, province Puerto Inca, department Huánuco, Peru. Target trees were pre-identified and subsequently submitted to molecular confirmation of identity with three markers (rbcL, psbA and trnL-F). The COI gene of 119 lepidopteran larvae was successfully sequenced and found to belong to 92 species: Comparison of DNA barcodes with the reference database of adult moths resulted in 65 (55%) matches at species level, 32 (27%) at genus level, 19 (16%) at subfamily or family level, three just to order level. Three larvae could not be assigned to a family. For these larvae the fogged target tree now suggests a potential host-plant relationship. Molecular gut content analysis, based on High-Throughput-Sequencing was successfully tested for ten larvae corroborating feeding on the target plant in some cases but elucidating several other cases of potential ‘alternative feeding’. We propose a larger-scale approach using this rapid and efficient method including molecular gut-content analyses for comprehensively testing the ratio of ‘alternative feeders’ and pitfalls caused by collateral fogging of larvae from neighboring trees.

## Introduction

Despite much valuable work on host-relationships of Neotropical moths, e.g. from Ecuador [1,2], or Costa Rica [3,4,5], the relevant literature is still scarce and patchy compared with the huge species diversity of Lepidoptera in Central and South America. Apart from the aforementioned programs only few original data are published for host-plant relationships of Lepidoptera and much of the work focused on caterpillars found on plants of economic importance (pests and potential pests) (e.g. [6,7,8]).

Exemplified from one of the most diverse moth families, Geometridae, the largest project in Costa Rica so far revealed the huge amount of 22,957 geometrid moth records, the barcoded reared adults clustering to 566 BINs, of which 162 currently having Linnean species names (D.J. Janzen & W. Hallwachs pers. comm.). Brehm [1] presented 48 neotropical geometrid species with host-plant records, with 11 records added by Dyer et al. [9] and 59 records by Bodner et al. [2]. Thus, altogether for some 680 Neotropical geometrid species (about 270 of which with Linnean species names) host-plant relationships are known, covering approx. 8–10% (4% with Linnean names) of the described geometrid fauna of Central and South America (for estimations of total number of described geometrid species cf. Scoble et al. [10]: 6433 species; Heppner [11]: 7956 species).

Estimation for Neotropical species diversity is based on approx. 37,000 described Neotropical moth species (Heppner [11]: 44,800 described Lepidoptera, including approx. 7800 Rhopalocera species [12]) and considering that (a) ‘Microlepidoptera’ are severely understudied and (b) the vast majority (>70%) of the Neotropical moth fauna is still undescribed as suggested by the ratio of undescribed species in some 380,000 Neotropical lepidopteran DNA barcodes on Barcode of Life Data Systems (‘BOLD’ [13]; accessed September 2019). Extrapolating the aforementioned data on species numbers and feeding records we estimate that for >98% of the putatively >100,000 Neotropical moth species authentic feeding records from nature are lacking.

Traditionally, most insect larvae are identified by rearing them to the adult stage and by analysing the morphology of the adult. Methodological constraints in this classic approach are (1) visual search and collecting on plant depending on the skills of the biologist, (2) the canopy region of trees hardly accessible, (3) nocturnal activity of many larvae requiring difficult search by night, (4) collecting without feeding observation may lead to misinterpretations ([14,15]: 20–50% „alternative feeders”on lichens, dead leaves, algae, etc), (5) beating, shaking, net-sweeping may obscure the real where-about of the larva, (6) feeding records in rearing may not reflect the natural host-plant association, (7) rearing to adult is time consuming, (8) rearing may fail (deseases, parasitoids), (9) identification and availability of host-plant (for rearing) often difficult.

Molecular identification of lepidopteran larvae and other insects through DNA barcoding (COI 5’) was repeatedly carried out successfully, e.g. [16,17,18,19,20], permitting an easy, cheap and rapid identification of larvae collected from their host-plants. Identification through DNA barcoding is possible even from dry skins after moulting and from empty pupal exuviae after hatching of the moths (own, unpublished data; [21]). Currently, there are large-scale projects devoted to the identification of larvae along with their host-plants in Papua New Guinea ([22]) and Costa Rica ([4]). Both are based on an integrative approach combining morphology, rearing and molecular techniques for the identification of the reared adults and/or their parasitoids.

Miller et al. [16] and Matheson et al. [17] investigated and ascertained relationships between plants and caterpillars through a method based on the DNA identification of the larval gut content, an effective but (in earlier times) expensive and time-consuming approach, especially as a routine application in larger surveys. Later on, molecular gut content analysis was proposed for unveiling insect-host plant associations e.g. for beetles [23,24,25,26], and for soil insects [27].

The aim of this pilot paper was to establish methodology to infer host-plant relationships of caterpillars based on the identification of larvae collected by insecticidal knock-down (canopy-fogging) on their food-plants through DNA barcoding and to use gut HTS-based content analysis to estimate potential pitfalls due to ‘alternative feeding’ or due to collateral fogging from neighbouring plants, lianas etc. (cf. Discussion).

## Material and methods

### Collecting and canopy fogging

Canopy fogging was performed by AmH und AF from the ground with a Swingfog SN 50 fogger, using natural Pyrethrum, diluted in a highly raffinated white oil, as knock-down agent to prevent the introduction of persistent chemicals into the environment. For details of the fogging procedure see [28]. In most cases, trees with dense foliage cover and little canopy overlap with neighboring trees were chosen. We made sure the fog reached the canopy and stood there for at least five minutes to affect the arthropods. In order to install the collecting sheets, larger saplings and other interferring vegetation elements were cleared below the tree projection area. All organisms dropping down from the trees were collected at least one hour after the fogging from expanded plastic sheets of 20 m^2^ size, covering an estimated minimum of 80% of the target tree canopy. All arthropods of each fogging event were pooled and then transferred into accurately labelled jars with 100% ethanol without pre-sorting. The following day the ethanol was renewed and excessive plant material with its high water content was removed. Samples were stored at room temperature for up to two weeks while in the research station Panguana. The ethanol was renewed again when the samples were added to the Zoologische Staatssammlung München in Bavaria, Germany (SNSB–ZSM).

The study site is located in the westernmost Amazonian Basin, eastern central Peru, department Huánuco, at the ACP Panguana station (-9.613393°N -74.935911°E; 222 m; see also [29]), the fogged target trees were all situated in a radius of less than 2000 meters around the station. Collecting was performed in the late afternoon, betwen 17 and 19 o’clock, from 24^th^ of November to 8^th^ of December 2017. For identification of the target trees see results.

Collection permits were released by Servicio Nacional Forestal y de Fauna Silvestre SERFOR: No. 007-2014-SERFOR-DGGSPFFS + No. 0406-2017-SERFOR-DGGSPFFS, export permits fauna: No. 003236-SERFOR; 003281-SERFOR; 003320-SERFOR; export permits flora: No. 003284-SERFOR + Resolution of General Direction No. 161-2018-MINAGRI-SERFOR-DGGSPFFS; No. 003333-SERFOR. Field site access was granted by SERFOR and Biological Research Station ACP Panguana. Further ethical approval was not required for the data analysis, since no in vivo experiments were performed.

### Tissue sampling and identification of larvae (DNA barcoding, COI)

**Fig 1:**
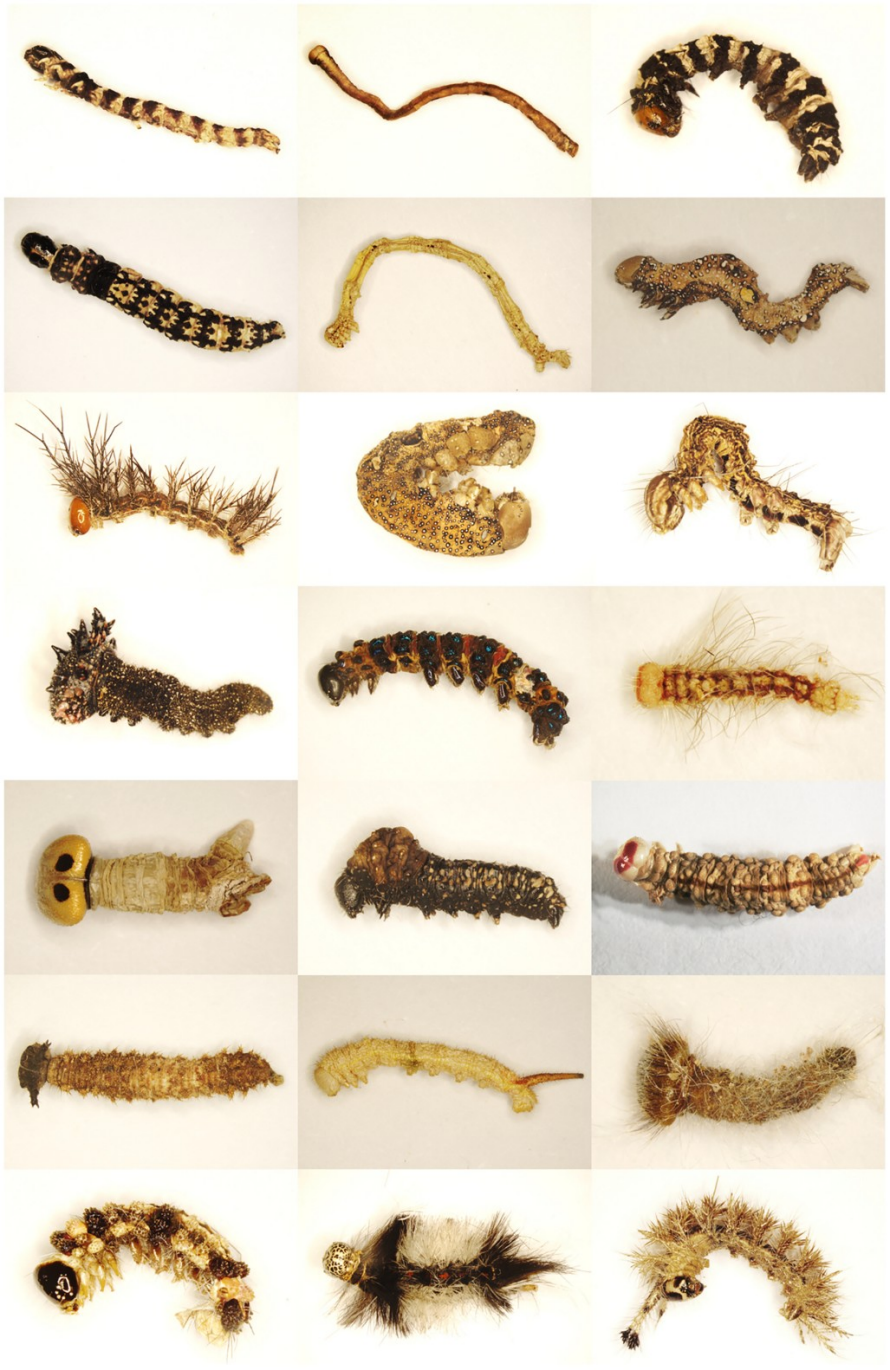
Lepidopteran larvae after selection from the alcohol-preserved fogging samples from Panguana, Peru, after drying and before tissue-sampling for the DNA analysis. (Upper) row 1: larvae nr. 5, 13, 26; row 2: nr. 28, 46, 52; row 3: nr. 62, 65, 71; row 4: nr. 81, 83, 85; row 5: nr. 88, 93, 100; row 6: nr. 109, 113, 115; (bottom) row 7: nr. 119, 122, 124 (numbers and identification of larvae see Table 2).

Out of 47 samples–each of them referring to a fogged tree (cf. Table 3 and S1 Table)–all lepidopteran larvae were separated, in total 130 specimens. The larvae were dried on paper, photographed and then separately stored in Eppendorf tubes. A list of all 130 larvae along with their fogging sample number is given in Table 1, examples are shown in Fig 1. Tissue sampling was carried out for all 130 larvae by using scissors and pincers, which were carefully cleaned after each tissue sampling in 100% alcohol followed by exposure to a burner to avoid contamination among samples. Tissues (one vertically cut segment, in very small larvae two segments) were transferred to a lysis plate, adding 0.5 ml of 100% alcohol to each well on the plate. On each plate one well was used for negative control.

**Table 1:** Overview on the 130 Lepidoptera larvae selected from 36 fogging samples from Panguana, Peru, sequencing success and number of target tree.

Larva Nr.	Barcode ID	Sequence Length (bp)	Target tree nr.
1	BC ZSM Lep 98047	658	2
2	BC ZSM Lep 98048	658	2
3	BC ZSM Lep 98049	658	2
4	BC ZSM Lep 98050	658	3
5	BC ZSM Lep 98051	658	7
6	BC ZSM Lep 98052	658	9
7	BC ZSM Lep 98053	658	9
8	BC ZSM Lep 98054	658	14
9	BC ZSM Lep 98055	658	14
10	BC ZSM Lep 98056	658	15
11	BC ZSM Lep 98057	658	16
12	BC ZSM Lep 98058	658	16
13	BC ZSM Lep 98059	658	19
14	BC ZSM Lep 98060	658	20
15	BC ZSM Lep 98061	635	20
16	BC ZSM Lep 98062	658	20
17	BC ZSM Lep 98063	658	21
18	BC ZSM Lep 98064	658	22
19	BC ZSM Lep 98065	658	22
20	BC ZSM Lep 98066	658	23
21	BC ZSM Lep 98067	658	23
22	BC ZSM Lep 98068	658	24
23	BC ZSM Lep 98069	658	24
24	BC ZSM Lep 98070	658	25
25	BC ZSM Lep 98071	0	27
26	BC ZSM Lep 98072	658	27
27	BC ZSM Lep 98073	658	27
28	BC ZSM Lep 98074	658	27
29	BC ZSM Lep 98075	658	28
30	BC ZSM Lep 98076	658	28
31	BC ZSM Lep 98077	658	29
32	BC ZSM Lep 98078	658	32
33	BC ZSM Lep 98079	658	32
34	BC ZSM Lep 98080	658	32
35	BC ZSM Lep 98081	658	32
36	BC ZSM Lep 98082	658	32
37	BC ZSM Lep 101897	658	1
38	BC ZSM Lep 101898	658	1
39	BC ZSM Lep 101899	658	1
40	BC ZSM Lep 101900	658	2
41	BC ZSM Lep 101901	0	2
42	BC ZSM Lep 101902	658	2
43	BC ZSM Lep 101903	658	5
44	BC ZSM Lep 101904	0	7
45	BC ZSM Lep 101905	658	7
46	BC ZSM Lep 101906	658	9
47	BC ZSM Lep 101907	658	9
48	BC ZSM Lep 101908	658	10
49	BC ZSM Lep 101909	658	10
50	BC ZSM Lep 101910	658	10
51	BC ZSM Lep 101911	658	10
52	BC ZSM Lep 101912	658	11
53	BC ZSM Lep 101913	658	12
54	BC ZSM Lep 101914	658	12
55	BC ZSM Lep 101916	658	13
56	BC ZSM Lep 101917	658	13
57	BC ZSM Lep 101918	0	13
58	BC ZSM Lep 101919	658	14
59	BC ZSM Lep 101920	658	14
60	BC ZSM Lep 101921	658	14
61	BC ZSM Lep 101922	658	14
62	BC ZSM Lep 101923	658	14
63	BC ZSM Lep 101924	658	14
64	BC ZSM Lep 101925	658	15
65	BC ZSM Lep 101926	658	15
66	BC ZSM Lep 101927	658	15
67	BC ZSM Lep 101928	658	17
68	BC ZSM Lep 101929	658	17
69	BC ZSM Lep 101930	658	17
70	BC ZSM Lep 101931	658	18
71	BC ZSM Lep 101932	658	19
72	BC ZSM Lep 101933	658	19
73	BC ZSM Lep 101934	658	19
74	BC ZSM Lep 101935	658	19
75	BC ZSM Lep 101936	658	19
76	BC ZSM Lep 101937	658	19
77	BC ZSM Lep 101938	658	19
78	BC ZSM Lep 101939	658	20
79	BC ZSM Lep 101940	658	20
80	BC ZSM Lep 101941	658	20
81	BC ZSM Lep 101942	658	20
82	BC ZSM Lep 101943	658	21
83	BC ZSM Lep 101944	658*	21
84	BC ZSM Lep 101945	658	22
85	BC ZSM Lep 101946	658	23
86	BC ZSM Lep 101947	658	23
87	BC ZSM Lep 101948	0	23
88	BC ZSM Lep 101949	658	23
89	BC ZSM Lep 101950	658	23
90	BC ZSM Lep 101951	658	23
91	BC ZSM Lep 101952	658	23
92	BC ZSM Lep 101953	658	23
93	BC ZSM Lep 101954	658	24
94	BC ZSM Lep 101955	0	25
95	BC ZSM Lep 101956	658	25
96	BC ZSM Lep 101957	658	27
97	BC ZSM Lep 101958	658	27
98	BC ZSM Lep 101959	658	28
99	BC ZSM Lep 101960	658	29
100	BC ZSM Lep 101961	658	29
101	BC ZSM Lep 101962	658	32
102	BC ZSM Lep 101963	658*	32
103	BC ZSM Lep 101964	658	32
104	BC ZSM Lep 101965	658	32
105	BC ZSM Lep 101966	658	32
106	BC ZSM Lep 101967	0	32
107	BC ZSM Lep 101968	658*	32
108	BC ZSM Lep 101969	658*	32
109	BC ZSM Lep 101970	658	32
110	BC ZSM Lep 101971	658	32
111	BC ZSM Lep 101972	658	33
112	BC ZSM Lep 101973	658	33
113	BC ZSM Lep 101974	658	34
114	BC ZSM Lep 101975	658	35
115	BC ZSM Lep 101976	658	35
116	BC ZSM Lep 101977	658	36
117	BC ZSM Lep 101978	658	36
118	BC ZSM Lep 101979	0	36
119	BC ZSM Lep 101980	658	37
120	BC ZSM Lep 101981	658	37
121	BC ZSM Lep 101982	658	38
122	BC ZSM Lep 101983	658	38
123	BC ZSM Lep 101984	658	40
124	BC ZSM Lep 101985	0	40
125	BC ZSM Lep 101986	0	40
126	BC ZSM Lep 101987	658	41
127	BC ZSM Lep 101988	658	42
128	BC ZSM Lep 101989	658	44
129	BC ZSM Lep 101990	0	44
130	BC ZSM Lep 101991	658	47

Identity of larva see Table 2, identity of trees see Table 3 and S1+S3 Tables;

* sequenced by AIM company with special primers for alcaloid-inhibited samples.

**Table 2:** Identification results from sequence blasting on BOLD for 119 successfully sequenced Lepidoptera larvae (see Table 1) and their distances from the nearest genetic neighbour.

Larva Nr.	Family	Identification of larvae from BOLD-blast	Nearest neighbour (NN) on BOLD	distance from NN (%)	category of match
1	Bombycidae	*Quentalia*	*Quentalia chromanaDHJ01*	5.8	genus
2	Bombycidae	*Quentalia*	*Quentalia chromanaDHJ01*	5.8	genus
3	Erebidae	*Lascoria*	*Lascoria* species indet.	0.45	species
4	Gelechiidae	Gelechiidae	Gelechiidae genus indet.	8.0	family
5	Gelechiidae	*Dichomeris*	*Dichomeris* species indet.	4.6	genus
6	Geometridae	Larentiinae	*Xanthorhoe labradorensis*	8.1	family
7	Plutellidae	Plutellidae	*Rhigognostis senilella*	8.9	family
8	Erebidae	*Ypsora selenodes*	*Ypsora selenodes*	1.2	species
9	Geometridae	*Thysanopyga apicitruncaria*	*Thysanopyga apicitruncaria*	0.15	species
10	Tineidae	*Hybroma*	*Hybroma* species indet.	6.9	genus
11	Crambidae	Spilomelinae_genus sp. 30YB	Spilomelinae_genus sp. 30YB	1.4	species
12	Crambidae	Spilomelinae_genus sp. 30YB	Spilomelinae_genus sp. 30YB	1.1	species
13	Geometridae	*Hemipterodes divaricata*	*Hemipterodes* BioLep111	3.5	genus
14	Erebidae	Erebidae	*Bertula tespisalis*	6.6	family
15	Erebidae	Erebidae	*Bertula tespisalis*	6.6	family
16	Erebidae Cte.	*Delphyre orientalis*	*Delphyre orientalis*	1.2	species
17	Riodinidae	Riodinidae	*Semomesia croesus*	7.5	family
18	Erebidae Lit.	*Prepiella* species 1	*Prepiella* species indet.	0.15	species
19	Gelechiidae	Gelechiidae	Gelechiidae genus indet.	2.4	genus
20	Geometridae	*Patalene hamulataAH01Pe*	*Patalene hamulataAH01Pe*	0.0	species
21	Depressariidae	Depressariidae	*Antaeotricha Janzen86*	7.5	family
22	Uraniidae	Uraniidae species indet.	Uraniidae species indet.	0.0	species
23	Uraniidae	Uraniidae species indet.	Uraniidae species indet.	0.0	species
24	Noctuidae	noctBioLep01 BioLep2008	noctBioLep01 BioLep2008	1.4	species
26	Uraniidae	*Urania leilus*	*Urania leilus*	0.0	species
27	Depressariidae	Depressariidae	*Stenoma* species indet.	7.9	family
28	Depressariidae	Depressariidae	*Stenoma* species indet.	7.9	family
29	Uraniidae	Uraniidae	*Cyphyra swinhoei* (Uran.)	7.2	family
30	Erebidae Lit.	*Nodozana* nr. *coresa*	*Nodozana* nr. *coresa*	0.15	species
31	Gelechiidae	Gelechiidae	GelJanzen01 Janzen180	7.8	family
32	Apatelodidae	*Olceclostera*	*Olceclostera* species indet.	6.9	genus
33	Depressariidae	Depressariidae	*Stenoma* species indet.	6.9	family
34	Depressariidae	Depressariidae	*Stenoma* species indet.	6.7	family
35	Gelechiidae	Dichomeris?	Gelechiidae genus indet.	2.2	species
36	Depressariidae	Depressariidae	Depressariidae genus indet.	2.6	genus
37	Noctuidae	Noctuidae_incertae_sedis sp. 14YB	Noctuidae_incertae_sedis sp. 14YB	1.9	species
38	Geometridae	*Ergavia*	*Ergavia* species indet.	3.8	genus
39	Notodontidae	Notodontidae	*Hemiceras plana*	9.4	family
40	Geometridae	*Physocleora* AH02Pe	*Physocleora* AH02Pe	0.15	species
42	Bombycidae	*Quentalia*	*Quentalia chromanaDHJ01*	5.8	genus
43	Hesperiidae	*Panoquina fusina*	*Panoquina fusina*	0.0	species
45	Gelechiidae	Gelechiidae	Gelechiidae genus indet.	8.5	family
46	Geometridae	Larentiinae species	Larentiinae genus indet.	0.15	species
47	Erebidae	*Mecodina*	*Mecodina* species indet.	8.1	genus
48	Apatelodidae	*Olceclostera*	*Olceclostera* species indet.	6.4	genus
49	Erebidae	*Deinopa*	*Deinopa angitia*	5.1	genus
50	unidentified	Lepidoptera	*Semomesia croesus* (Riodin.)	9.6	order
51	unidentified	Lepidoptera	Semomesia croesus (Riodin.)	9.8	order
52	Erebidae	*Eudocima procus*	*Eudocima procus*	0.0	species
53	Erebidae	*Gorgone umbrigensDHJ02*	*Gorgone umbrigensDHJ02*	0.6	species
54	unidentified	Lepidoptera	*Semomesia croesus* (Riodin.)	9.6	order
55	Crambidae	*Evergestis*	*Evergestis simulatilis*	6.1	genus
56	Erebidae	Erebidae	*Catocala retecta*	6.6	family
58	Geometridae	*Perissopteryx divisaria*	*Perissopteryx divisaria*	1.4	species
59	Geometridae	*Thysanopyga apicitruncaria*	*Thysanopyga apicitruncaria*	0.15	species
60	Erebidae	*Lascoria* Poole03	*Lascoria* Poole03	0.45	species
61	Erebidae	*Letis magna*	*Letis magna*	0.0	species
62	Saturniidae	*Automeris denticulata*	*Automeris denticulata*	0.0	species
63	Bombycidae	*Anticla*	*Anticla anticaDHJ04*	2.9	genus
64	Erebidae	*Eudocima procus*	*Eudocima procus*	0.0	species
65	Erebidae	*Eudocima procus*	*Eudocima procus*	0.0	species
66	Erebidae Phae.	*Ernassa* species	*Ernassa* species indet.	2.1	species
67	Phiditiidae	*Phiditia*	*Phiditia lucernaria*	3.8	genus
68	Geometridae	*Pero incisa*	*Pero incisa*	0.15	species
69	Erebidae	*Lascoria* Poole03	*Lascoria* Poole03	0.45	species
70	Erebidae	*Metalectra*	*Metalectra* BioLep167	5.2	genus
71	Erebidae	*Latebraria amphipyroides*	*Latebraria amphipyroides*	0.6	species
72	Noctuidae	*Drobeta*	*Drobeta* Poole17	4.1	genus
73	Erebidae	*Latebraria amphipyroides*	*Latebraria amphipyroides*	0.6	species
74	Geometridae	*Idaea orilochia*	*Idaea orilochia*	0.0	species
75	Geometridae	*Hemipterodes divaricata*	*Hemipterodes* BioLep111	3.5	genus
76	Apatelodidae	Apatelodidae	Apatelodidae genus indet.	6.6	family
77	Erebidae	*Mastixis*	*Mastixis* Poole02	3.5	genus
78	Erebidae	*Feigeria scops*	*Feigeria scops*	0.0	species
79	Geometridae	Sterrhinae	*Cyclophora* species indet.	6.1	family
80	Geometridae	*Semaeopus*	*Semaeopus* Janzen216	5.5	genus
81	Nymphalidae	*Memphis acidalia*	*Memphis acidalia*	0.0	species
82	Euteliidae	*Paectes*	*Paectes circularis*	3.7	genus
83	Erebidae Cte.	*Calonotos chalcipleura* (Ereb.)	*Calonotos chalcipleura*	0.0	species
84	Erebidae Lit.	*Clemensia*	Erebidae genus indet. (*Clemensia*)	1.7	species
85	Erebidae Lit.	*Prepiella* species 2	*Prepiella* species indet.	0.3	species
86	Geometridae	*Patalene hamulataAH01Pe*	*Patalene hamulataAH01Pe*	0.0	species
88	Hesperiidae	*Polythrix*	*Polythrix kanshul*	5.3	genus
89	Erebidae Phae.	*Stidzaeras strigifera*	*Stidzaeras strigifera*	2.2	species
90	Erebidae Lit.	*Apistosia judas*	*Apistosia judas*	0.0	species
91	Erebidae Phae.	*Stidzaeras strigifera*	*Stidzaeras strigifera*	1.9	species
92	Saturniidae	*Pseudautomeris arminirene*	*Pseudautomeris arminirene*	0.8	species
93	Uraniidae	Uraniidae species indet.	Uraniidae species indet.	0.0	species
95	Erebidae	*Lascoria manes*	*Lascoria manes*	0.8	species
96	Erebidae	*Clapra*	*Clapra* species indet	2.1	species
97	Erebidae	*Lascoria* Poole03	*Lascoria* Poole03	0.0	species
98	Saturniidae	*Homoeopteryx*	*Homoeopteryx major*	2.7	genus
99	Erebidae Cte.	*Haemanota nigricollum*	*Haemanota nigricollum*	0.0	species
100	Erebidae Cte.	*Haemanota nigricollum*	*Haemanota nigricollum*	0.0	species
101	Notodontidae	*Kaseria*	*Kaseria pallida*	4.6	genus
102	Geometridae	*Mychonia* (Geom.)	*Mychonia*	0.0	species
103	Noctuidae	*Lycaugesia*	Noctuidae genus indet. (*Lycaugesia*)	3.2	genus
104	Geometridae	*Ischnopteris chlorophaearia*	*Ischnopteris chlorophaearia*	0.0	species
105	Apatelodidae	*Olceclostera*	*Olceclostera* species indet.	2.6	genus
107	Erebidae	*Lascoria* Poole03 (Ereb.)	*Lascoria* Poole03	0.0	species
108	Erebidae Lit.	*Apistosia judas* (Ereb.)	*Apistosia judas*	0.0	species
109	Nymphalidae	*Euptychia* n. sp. 5 CP-2006	*Euptychia* n. sp. 5 CP-2006	1.6	species
110	Apatelodidae	Apatelodidae species	Apatelodidae genus indet.	2.0	species
111	Erebidae	*Antiblemma sterope*	*Antiblemma sterope*	0.5	species
112	Erebidae	*Eudocima*	*Eudocima* species indet.	6.1	genus
113	Bombycidae	*Anticla*	*Anticla anticaDHJ03*	6.6	genus
114	Erebidae	*Sosxetra grata*	*Sosxetra grata*	0.0	species
115	Hesperiidae	*Myscelus epimachia*	*Myscelus epimachia*	0.0	species
116	Geometridae	*Glena* AH03Pe	*Glena* AH03Pe	0.15	species
117	Geometridae	*Semiothisa gambaria*	*Semiothisa gambaria*	0.0	species
119	Erebidae Phae.	*Melese*	*Melese drucei*	3.2	genus
120	Geometridae	*Physocleora* AH02Pe	*Physocleora* AH02Pe	0.3	species
121	Geometridae	*Stegotheca*	*Stegotheca* species indet.	3.5	genus
122	Erebidae Phae.	*Pelochyta arontes*	*Pelochyta arontes*	0.0	species
123	Geometridae	*Stegotheca*	*Stegotheca* species indet.	3.2	genus
126	Erebidae	*Sosxetra grata*	*Sosxetra grata*	0.0	species
127	Noctuidae	Noctuidae	Noctuidae/Acontiinae genus indet.	4.0	genus
128	Erebidae	Erebidae	*Phytometra ernestinana*	8.5	family
130	Geometridae	*Stegotheca*	*Stegotheca* species indet.	3.5	genus

Lit. = Lithosiini (Arctiinae); Cte. = Ctenuchina (Arctiinae); Phae. = Phaegopterina (Arctiinae)

Tissue samples were submitted to the standard procedures of the Canadian Centre for DNA Barcoding (CCDB) for sequencing the mitochondrial 5’ cytochrome oxidase gene, subunit 1 (COI), the standard marker for the identification of most animals. LepF1 and LepR1 were the primers used for PCR and sequencing [30]. Sequences were blasted against the complete sequence database of the Barcode of Life Data systems (BOLD, [13]) in order to infere the closest matches using the BOLD Identification Engine (http://www.boldsystems.org/index.php/ IDS_OpenIdEngine). Also morphology of larvae and related (genetically near) adult moths were considered to test the reliability of the results. Nomenclature of scientific taxon names follows the catalogue used on BOLD database, which in many families is in accordance with the currently available catalogues (e.g. [31] for Geometridae). Vouchers of larvae are stored at the Zoologische Staatssammlung München, Germany. Sequences, images and related metadata are available open access on BOLD under the dataset DS-PANLARVA (dx.doi.org/10.5883/DS-PANLARVA).

### Tissue sampling and morphology-based identification of target trees

The 47 target trees have been pre-identified in the field based on morphology (shape of tree growth and shape of leaves, rarely blossoms or fruits) by the native caretaker of the Panguana Station, “Moro” Carlos Vásquez Módena, to Peruvian vernacular names (see Table 3 and S1 Table), which usually cannot be unequivocally referred to scientific plant names, however. For a tentative assignment of vernacular names to botanical taxa see S1 Table. For nomenclature of plant names we follow the “Plant List” (available online at www.theplantlist.org/1/). For most target trees a small branch was collected, pressed and kept in a herbarium for identification. Identification of a selection of sampled leaves was performed by Hamilton Paredes, Museo de Historia Natural, Lima. A small leaf piece was cut as tissue sample for DNA-Barcoding. In addition to that, sapwood/cambium tissue samples were taken of each target tree by using a leather punch to extract a core from the stem. Then, a thin slice of sapwood/cambium was cut and immediately dried over silica gel.

**Table 3:** Identification of target trees, results from blasting on NCBI (BLAST matches usually >99%).

Target tree nr.	Preidentification (from vernacular names; cf. S1 Table)	Molecular consensus identification (rbcL, trnL-F and psbA genes; cf. S3 Table)	Consensus identification
1	*Mangifera indica*	*Mangifera indica*	*Mangifera indica* (Anacardiaceae)
2	*Mangifera indica*	*Mangifera indica*	*Mangifera indica* (Anacardiaceae)
3	Meliaceae or Annonaceae	*Guarea* or *Cabralea* (Meliaceae)	*Guarea* or *Cabralea* (Meliaceae)
4 #	Anacardiaceae	*Mangifera* or *Spondias* (Anacardiaceae)	*Mangifera* or *Spondias* (Anacardiaceae)
5	*Guarea* (Meliaceae)	*Guarea* or *Cabralea* (Meliaceae)	*Guarea* (Meliaceae)
6 #	*Ficus* (Moraceae)	*Ficus* (Moraceae)	*Ficus* (Moraceae)
7	‚Ucu muchaca‘ ^1^	Malvaceae or Meliaceae ^2^	Malvaceae or Meliaceae ^2^
8 #	*Apeiba* (Malvaceae)	Malvaceae	*Apeiba* (Malvaceae)
9	*Leonia glycycarpa* (Violaceae)	*Leonia glycycarpa* (Violaceae)	*Leonia glycycarpa* (Violaceae)
10	Annonaceae	*Oxandra polyantha* (Annonaceae)	*Oxandra polyantha* (Annonaceae)
11–1	*Celtis schippii* (Cannabaceae)	*Celtis schippii* (Cannabaceae)	*Celtis schippii* (Cannabaceae)
11–2	*Neea* (*Guapira*) (Nyctaginaceae)	*Neea* (Nyctaginaceae)	*Neea* (Nyctaginaceae)
12	Annonaceae	*Oxandra polyantha* (Annonaceae) and/or *Conceveiba guianensis* (Euphorbiaceae) ^2^	*Oxandra polyantha* (Annonaceae) and/or *Conceveiba guianensis* (Euphorbiaceae) ^2^
13	Annonaceae	*Oxandra polyantha* (Annonaceae)	*Oxandra polyantha* (Annonaceae)
14	*Poulsenia armata* (Moraceae)	*Naucleopsis* (Moraceae)	*Poulsenia* or *Naucleopsis* (Moraceae)
15	‚Ucu muchaca’ ^1^	*Hirtella* (Chrysobalanaceae)	*Hirtella* (Chrysobalanaceae)
16	*Castilla*	*Castilla elastica* (Moraceae)	*Castilla elastica* (Moraceae)
17	Moraceae	*Clarisia biflora* (Moraceae)	*Clarisia biflora* (Moraceae)
18	*Ficus* (Moraceae)	*Ficus* (Moraceae)	*Ficus* (Moraceae)
19	Annonaceae	*Oxandra polyantha* (Annonaceae)	*Oxandra polyantha* (Annonaceae)
20	no name provided	*Neea* (Nyctaginaceae)	*Neea* (Nyctaginaceae)
21	*Apeiba* (Malvaceae)	*Annona* (Annonaceae)	*Annona* (Annonaceae) or *Apeiba* sp.(Malvaceae)
22	‚Kaimitio‘	*Byrsonima coccolobifolia* (Malpighiaceae)	*Byrsonima coccolobifolia* (Malpighiaceae)
23	no name provided	no tissue provided	unidentified
24	*Perebea* (Moraceae)	*Pouteria* or *Chrysophyllum* (Sapotaceae)	Moraceae or Sapotaceae ^3^
25	*Otoba parvifolia* (Myristicaceae)	Myristicaceae	*Otoba parvifolia* (Myristicaceae)
26 #	*Apeiba* (Malvaceae)	Malvaceae	*Apeiba* (Malvaceae)
27	Annonaceae	*Oxandra polyantha* (Annonaceae)	*Oxandra polyantha* (Annonaceae)
28	*Ficus* (Moraceae)	*Ficus* (Moraceae) and/or *Simira* (Rubiaceae) ^2^	*Ficus* (Moraceae) and/or *Simira* (Rubiaceae) ^2^
29	*Apeiba* (Malvaceae)	Malvaceae	*Apeiba* (Malvaceae)
30 #	*Garcinia* (Clusiaceae)	*Garcinia macrophylla or G*. *mangostana* (Clusiaceae)	*Garcinia macrophylla or G*. *mangostana* (Clusiaceae)
31–1 #	*Garcinia* (Clusiaceae)	*Garcinia* (Clusiaceae)	*Garcinia* (Clusiaceae)
31–2 #	‚Tawari‘	Sapotaceae or Fabaceae ^2^	Sapotaceae or Fabaceae ^2^
32	Sapindaceae	*Paullinia* (Sapindaceae)	*Paullinia* (Sapindaceae)
33	*Guarea* (Meliaceae) ^1^	*Trichilia* (Meliaceae)	*Trichilia* (Meliaceae)
34	Moraceae	*Ficus* (Moraceae)	*Ficus* (Moraceae)
35	*Guarea* (Meliaceae) ^1^	*Guarea guidonia* (Meliaceae)	*Guarea guidonia* (Meliaceae)
36	*Guarea* (Meliaceae) ^1^	*Guarea guidonia* (Meliaceae)	*Guarea guidonia* (Meliaceae)
37	*Guarea* (Meliaceae) ^1^	*Guarea guidonia* (Meliaceae)	*Guarea guidonia* (Meliaceae)
38	*Guarea* (Meliaceae) ^1^	*Erythrina speciosa* (Fabaceae) ^2^	*Guarea* (Meliaceae) or *Erythrina* (Fabaceae) ^1^ ^2^
39 #	*Tapirira guianensis* (Anacardiac.)	*Tapirira guianensis* (Anacardiaceae) or *Guarea guidonia* (Meliaceae) ^1^ ^2^	*Tapirira guianensis* (Anacardiaceae) or *Guarea guidonia* (Meliaceae) ^1^ ^2^
40	*Guarea* (Meliaceae) ^1^	*Guarea guidonia* (Meliaceae)	*Guarea guidonia* (Meliaceae)
41	*Guarea* (Meliaceae) ^1^	*Guarea guidonia* (Meliaceae)	*Guarea guidonia* (Meliaceae)
42	*Ficus* (Moraceae)	*Ficus* (Moraceae)	*Ficus* (Moraceae)
43 #	*Guarea* (Meliaceae) ^1^	*Guarea guidonia* (Meliaceae)	*Guarea guidonia* (Meliaceae)
44	*Guarea* (Meliaceae) ^1^	*Guarea guidonia* (Meliaceae)	*Guarea guidonia* (Meliaceae)
45 #	*Guarea* (Meliaceae) ^1^	*Guarea guidonia* (Meliaceae)	*Guarea guidonia* (Meliaceae)
46 #	*Guarea* (Meliaceae) ^1^	*Guarea guidonia* (Meliaceae)	*Guarea guidonia* (Meliaceae)
47	*Guarea* (Meliaceae) ^1^	*Guarea guidonia* (Meliaceae)	*Guarea guidonia* (Meliaceae)

^#^ = fogging sample from target tree without lepidopteran larva;

^1^ = same pre-identified vernacular name with two different molecular identifications.

^2^ = potential sampling error (tissue sampling from two neighboring trees/plants);

^3^ = misidentified vernacular name or sampling error (tissue sampling from two neighboring trees/plants)

### Identification of target trees through DNA barcoding (trnL-F, rbcL & psbA)

Because of the above mentioned uncertainties of target tree pre-identification, we have submitted plant tissue samples to DNA barcoding. For that purpose leaves were available for 37 out of the 47 target trees, pieces of cambium+sapwood for 46 trees. Plant tissues (leaves) were submitted to Sanger sequencing (AIM; Advanced Identification Methods GmbH– www.aimethods-lab.com) with two markers, rbcL and psbA using standardized protocols following [32,33]. An additional attempt was performed in CCDB (Guelph, Canada; primers: trnL-F, rbcL; standard Sanger sequencing procedure) using both leaves and sapwood samples, the latter supplementing those cases where no leaves were available for study. The third marker (trnL-F) was added for further resolving the identification to genus and species level in some cases. All resulting sequences were blasted against GenBank (NBCI) and BOLD data using standard blast functions. Sequences and related metadata are available open access on BOLD under the dataset DS-PANPLANT (dx.doi.org/10.5883/DS-PANPLANT).

### Gut content analysis (rbcL, psbA)

For a subset of ten larvae, gut content analysis was tested for molecular identification of the larva’s ‘true’ diet. For that purpose, we performed a second vertical cut and submitted one segment of the larva to High-Throughput-Sequencing (HTS) with the two markers rbcL and psbA. Cut slices of the caterpillars were dried, homogenized and DNA extracted using the DNEasy Plant kit (Qiagen, Hilden, Germany). From each sample, 5 μL of extracted genomic DNA was used, along with plant TAQ (Bioline, Luckenwalde, Germany), and High Throughput Sequencing (HTS) adapted mini-barcode primers (trnH-psbA-f 5’-CGC GCA TGG TGG ATT CAC AAT CC-3’, trnH-psbA 5’-GTT ATG CAT GAA CGT AAT GCT-3’, [32,33], using the PCR conditions 95°C-4’– 35x 94°C-30”/55°C-30”/72°C-1’– 72°C-10’) were applied for PCR. Amplification success and fragment length were observed using gel electrophoresis. Amplified DNA was cleaned up and resuspended in 50 μL molecular water for each sample before proceeding. Successfully amplified products were used for a subsequent PCR reaction which adds Illumina Nextera XT indices to each PCR product, enabling a unique tagging of each sample. Illumina Nextera XT (Illumina Inc., San Diego, USA) indices were ligated to the samples in a second PCR reaction applying the same annealing temperature as for the first PCR reaction but with only seven cycles, and ligation success confirmed by gel electrophoresis (for detailed protocols see [34,35]). DNA concentrations were measured using a Qubit fluorometer (Life Technologies, Carlsbad, USA), and samples were combined into 40 μL pools containing equimolar concentrations of 100 ng each. Pools were loaded into a 1% agarose gel, run at 90 V for 45 minutes, bands of the target amplicon size were excised with sterilized razor blades, and purified with a GeneJet Gel Extraction kit (Life Technologies, Carlsbad, USA), following the manufacturer’s instructions. A final elution volume of 20 μL was used. High-Throughput Sequencing (HTS) was performed on an Illumina MiSeq using v2 (2*250 bp, 500 cycles, maximum of 20 mio. reads) chemistry. Negative controls for DNA metabarcoding analyses consisted of one negative-control-extraction (an empty DNEasy plant kit tube was extracted among the remaining ten caterpillar gut samples), one PCR negative control for each amplicon and one ligation negative control for each set of amplicons during library preparation with Nextera XT indices. Negative controls have been used to remove all OTUs with N(reads)< = 5 x sum of reads in negative controls (where sum of negative control reads is more than 20% of the number of reads in actual samples). All samples for each amplicon were separately pooled using equimolar amounts of 100 ng each. All samples were loaded on a single v2 2x250 bp MiSeq flow cell among other samples. Final DNA concentrations of amplicon pools were set to 380,000 total raw reads (190,000 paired end reads). The final concentration of the full library was 1.4 ng. Metabarcoding data are deposited and accessible on GenBank, BioProject ID PRJNA593715 (http://www.ncbi.nlm.nih.gov/bioproject/593715).

## Results

### Identification of larvae

A total of 130 caterpillar specimens were collected from 37 of the 47 target plants. No lepidopteran larvae were found in the samples of ten target trees. COI sequencing (DNA barcoding) was successful for 119 larvae (91.5%). The larvae belong to 92 different COI clusters (BINs), which are a good proxy for different species [36].

When blasting the DNA barcodes of the larvae on BOLD database, 65 larvae (55%) belonging to 48 species showed ‘close genetic similarity’–here defined as lower than 2.5%–with adult reference vouchers. Such genetic similarity is interpreted here as ‘species (or sister species) level matches’ (Table 2). 27 species have Linnean names on BOLD database, 20 are listed under ‘interim names’ (name codes) which either refer to described but not-yet-identified taxa or to undescribed species.

For 32 larvae (27%) belonging to 27 species the blasting on BOLD database revealed genus level matches, in five cases with disputable reliability. For 19 larvae (16%) assignment to subfamily or family level was possible, the reliability of 12 of these assignments needs to be tested by further extension of the reference database, since long branch attraction effects may have influenced the results in a few single cases. In just three cases belonging to one single species no family suggestion could be given based on the COI barcode.

### Identification of target plants

Pre-identification of target trees, as performed by the local administrator of the Panguana station (see S1 Table), was supplemented by molecular identification (sequencing of leaves and sapwood with the markers trnL-F, rbcL and psbA) of all but one of the target trees. For all but one of the target trees (98%) molecular identification through blasting on BOLD and GenBank brought a reliable identification to at least family level (see Table 3 and S3 Table). In four cases, however, the analysis of leaf and sapwood pointed to two different families which apparently is due to sampling errors, taking leaf and sapwood from different, neighbouring plants. In 37 target trees (79%) identification was possible to genus or species level (see Table 3 and S3 Table).

### Gut content analysis

Gut content analysis was performed for ten larvae based on Next-Generation-Sequencing with two markers rbcL and psbA. The two highest numbers of HTS-reads for rbcL and psbA genes and their genetically most similar species as resulting from BLAST-search in GenBank is shown for each larva in Table 4.

**Table 4:** Gut contents of ten fogged larvae with identity of target tree and HTS results from molecular identification of gut content, only the BLAST identification of the fragments with the two most numerous reads shown.

Nr. and identity of larva	Nr. and identity of target tree	HTS gut content (best hit): r(bcL), p(sbA)	nr. of reads	HTS gut content (second best hit)	nr. of reads
40 *Physocleora* AH02Pe (Geom.)	2 *Mangifera indica* (Anac.)	*Mangifera indica* (Anac.) r+p	12076	*Toxicodendron pubescens* (Anac.) r	1115
42 *Quentalia* (Bomb.)	3 *Guarea or Cabralea* (Meli.)	*Trophis racemosa* (Mora.) r	27876	(contaminations)	1–526
65 *Eudocima procus* (Ereb.)	15 *Hirtella* (Chrys.)	*Tinospora smilacina* (Meni.) ^1^ r	92678	*Odontocarya tamoides* (Meni.) ^1^ r	78516
76 Apatelodidae	19 *Oxandra polyantha* (Anno.)	*Cucumis sativus* (Cucu.) r	39129	*Lasthenia californica* (Aste.) r	37725
82 *Paectes* nr *circularis* (Noct.)	21 *Annona* (Anno.) or *Apeiba* (Malv.)	*Lejeunea bidentula* (Bryo.) p	6196	*Lejeunea tuberculosa* (Bryo.) p	5967
83—*Calonotos chalcipleura* (Ereb.)	21 *Annona* (Anno.) or *Apeiba* (Malv.)	*Echites yucatanensis* (Apoc.) ^2^ p	73311	*Anodendron* cf. *affine* (Apoc.) ^3^ p	68464
98 *Homoeopteryx* nr *major* (Satu.)	28 *Ficus* (Mora.) and/or *Simira* (Rubi.)!	*Faramea occidentalis* (Rubi.) r	109687	*Faramea pedunculata* (Rubi.) r	92548
102—*Mychonia* (Geom.)	32 *Paullinia* (Sapi.)	*Ceratolejeunea diversicornua* (Bryo.) ^5^ p	925060	*Schizocolea linderi* (Rubi.) ^4^ p	64812
107—*Lascoria* Poole03 (Ereb.)	32 *Paullinia* (Sapi.)	*Lejeunea bidentula* (Bryo.) ^5^ p	1550587	*Schizocolea linderi* (Rubi.) ^4^ p	395941
108—*Apistosia judas* (Ereb.)	32 *Paullinia* (Sapi.)	*Schizocolea linderi* (Rubi.) ^4^ p	934090	*Nyholmiella obtusifolia* (Bryo.) p	482948

p = psbA; r = rbcL.

^1^ liana, Australian;

^2^ liana, South American;

^3^ Asian;

^4^ African;

^5^ with several other sub-optimal blast hits in Bryophyta.

Abbreviations of lepidopteran families: Geom. = Geometridae; Bomb. = Bombycidae; Ereb. = Erebidae; Noct. = Noctuidae; Satu. = Saturniidae. Abbreviations of plant families: Anac. = Anacardiaceae; Mora. = Moraceae; Meli. = Meliaceae; Chry. = Chrysobalanaceae; Anno. = Annonaceae; Malv. = Malvaceae; Rubi. = Rubiaceae; Sapi. = Sapindaceae; Meni. = Menispermaceae; Cucu. = Cucurbitaceae; Bryo. = Bryophyta; Apoc. = Apocynaceae; Aste. = Asteraceae.

## Discussion

When investigating host-plant relationships it is usually assumed that larvae feed on the plants from where they have been collected. This assumption is based on the behaviour of larvae usually resting on their feeding plant during their development. One needs to consider, however, that certain larvae abandon their host-plants searching for a hidden resting place during daytime and mature larvae often leave their food-plant in the last days before pupation looking for a suitable pupation site, sometimes far from their feeding plants. Moreover, in particular in rainwood forests “alternative feeders” may use epiphytes, lianas, lichens, algae, fungi or mosses [14,15], and in our fogging approach pitfalls are possible through collateral fogging of larvae from neighboring trees. Gut content analysis can shed light on true feeding biology.

### Gut content matching identity of target tree

Only in one out of ten analysed larvae (see Table 4) the gut content revealed to match exactly the fogged target tree species: *Physocleora* AH02Pe (Geometridae; larva nr. 40) fogged from *Mangifera indica*. In a second case, *Homoeopteryx* near *major* (Saturniidae; larva nr. 98), the gut content revealed to be from the same plant family (Rubiaceae), genus *Simira* resulting from sequencing of plant sapwood and genus *Faramea* resulting from gut content analysis. The high percentage of eight out of ten larvae with a mismatch between target tree and gut content suggests that an a priori assignation of fogged larvae to the target trees usually is erroneous and that alternative feeding (epiphytes, algae, mosses etc.) or feeding on lianas and neighboring trees plays a major role. The rate of alternative feeding should be tested basing on a larger sample, ruling out a potentially biased ratio through external contamination of larvae by plant DNA (see below).

### Gut content matching previously known host-plant but not the target tree

A larva of the genus *Quentalia* (Bombycidae, larva nr. 42, see Table 4) was fogged from a tree of the family Meliaceae, but the gut content pointed to feeding on *Trophis racemosa* (Moraceae). Since *Quentalia* larvae were previously recorded as feeding on Moraceae [4], *Trophis racemosa* is likely the true food-plant of the *Quentinalia* larva which may have been growing close to the target tree.

In a second case, *Eudocima procus* (Erebidae; larva nr. 65, see Table 4) was fogged from a tree of the genus *Hirtella* (Chrysobalanaceae) but the gut content pointed to feeding on *Tinospora smilacina* (Menispermaceae). Since species of the genus *Eudocima* are known to feed on Menispermaceae [4,37,38], *Tinospora smilacina* is likely the true food-plant of the *Eudocima* larva. *Tinospora* is a liana and likely was associated with the target tree.

A similar case is also referring to liana-feeding: larva nr. 83 (see Table 4) was fogged from a tree of Annonaceae or Malvaceae, but in its gut content we found the DNA of the neotropical liana *Echites yucatanensis* (Apocynaceae).

Hence in three out of ten cases (30%) feeding on lianas or on a neighboring tree was recorded. Although the rate of feeding on such associated or neighbouring plants should be tested basing on a larger sample, the results of this pilot study clearly show that an *ad hoc* correlation of target tree and feeding biology is often premature and incorrect.

### Gut content not matching target tree but potentially pointing to alternative feeding

In four cases (larvae nr. 82, 102, 107, 108, see Table 4) the larvae were fogged down from trees (genus *Paullinia*, Sapindaceae; genus *Annona*, Annonaceae; genus *Apeiba*, Malvaceae), but the cut content was pointing to alternative feeding on mosses (Bryophyta). In the case of the *Lascoria* species (Erebidae; larva nr. 107) such alternative feeding is not excluded as larvae of this species were already observed when grazing on algae in Costa Rica [4]. However, moss-feeding is very unusual in Lepidoptera, and this may also be caused by contamination since these fogging samples (under 80% alcohol) contained some leaves of *Lejeunia* mosses whose DNA may have invaded the larvae through their stigmata or contaminated them on their skin. Further research is needed to estimate the influence of contamination through the sample alcohol. For this purpose larvae should be de-contaminated by bleeching before sequencing. In addition, their gut content could be extracted carefully by cutting the larva longitudinally.

### Inferring potential hostplant relationships (larvae without gut content analysis)

43 larvae with reliable identification to at least genus level, fogged from trees identified to at least genus level give first ‘hints’ on potential host-plants (Table 5). Almost all of them are new records, none of them was found in the ‘Hosts’ database [39] nor in Janzen & Hallwachs [4]. Alternative feeding, however, is not excluded (see notes to larvae nr. 18, 38, 43, 74 and 84 in Table 5), hence all suggested host-plant relationships require confirmation.

**Table 5:** Potential host-plant relationships for 43 larvae identified to at least genus level and identity of the fogged target tree.

Nr. of larva(e)	Identificarion of larva	Nr. of target tree(s)	Family of target tree	Identity of target tree
	**Nymphalidae**			
81	*Memphis acidalia*	20	Nyctaginaceae	*Neea*
109	*Euptychia* n. sp. 5 CP-2006	32	Sapindaceae	*Paullinia*
	**Hesperiidae**			
43	*Panoquina fusina* (1)	5	Meliaceae	*Guarea*
115	*Myscelus epimachia*	35	Meliaceae	*Guarea guidonia*
	**Apatelodidae**			
105	*Olceclostera*	32	Sapindaceae	*Paullinia*
	**Saturniidae**			
62	*Automeris denticulata*	14	Moraceae	*Poulsenia/Naucleopsis*
	**Bombycidae**			
63	*Anticla* near *anticaDHJ04*	14	Moraceae	*Poulsenia/Naucleopsis*
113	*Anticla* near *anticaDHJ03*	34	Moraceae	*Ficus*
	**Geometridae**			
13, 75	*Hemipterodes divaricata*	19	Annonaceae	*Oxandra polyantha*
74	*Idaea orilochia*	19	Annonaceae	*Oxandra polyantha*
38	*Ergavia*	1	Anacardiaceae	*Mangifera indica*
9, 59	*Thysanopyga apicitruncaria*	14	Moraceae	*Poulsenia/Naucleopsis*
58	*Perissopteryx divisaria*	14	Moraceae	*Poulsenia/Naucleopsis*
68	*Pero incisa*	17	Moraceae	*Clarisia biflora*
104	*Ischnopteris chlorophaearia*	32	Sapindaceae	*Paullinia*
116	*Glena* AH03Pe	36	Meliaceae	*Guarea guidonia*
120	*Physocleora* AH02Pe	37	Meliaceae	*Guarea guidonia*
117	*Semiothisa gambaria*	36	Meliaceae	*Guarea guidonia*
123, 130	*Stegotheca*	40, 47	Meliaceae	*Guarea guidonia*
	**Uraniidae**			
26	*Urania leilus*	27	Annonaceae	*Oxandra polyantha*
	**Noctuidae**			
103	*Lycaugesia*	32	Sapindaceae	*Paullinia*
	**Erebidae Arctiinae**			
16	*Delphyre orientalis*	20	Nyctaginaceae	*Neea*
18	*Prepiella* species 1 (4)	22	Malpighiaceae	*Byrsonima coccolobifolia*
66	*Ernassa*	15	Chrysobalanaceae	*Hirtella*
84	*Clemensia* species 1 (4)	22	Malpighiaceae	*Byrsonima coccolobifolia*
99, 100	*Haemanota nigricollum*	29	Malvaceae	*Apeiba*
119	*Melese*	37	Meliaceae	*Guarea*
	**Erebidae other subfamilies**			
8	*Ypsora selenodes*	14	Moraceae	*Poulsenia/Naucleopsis*
61	*Letis magna*	14	Moraceae	*Poulsenia/Naucleopsis*
69	*Lascoria* Poole03	17	Moraceae	*Clarisia biflora*
95	*Lascoria manes*	25	Myristicaceae	*Otoba parvifolia*
71, 73	*Latebraria amphipyroides*	19	Annonaceae	*Oxandra polycarpa*
77	*Mastixis*	19	Annonaceae	*Oxandra polycarpa*
78	*Feigeria scops*	20	Nyctaginaceae	*Neea*
96	*Clapra*	27	Annonaceae	*Oxandra polycarpa*
111	*Antiblemma sterope*	33	Meliaceae	*Trichilia*
114, 126	*Sosxetra grata*	35, 41	Meliaceae	*Guarea*

^(1)^ potential alternative feeding (members of genus *Panoquina* are known as feeders on monocotyledon plants like Poaceae);

^(2)^ potential alternative feeding (members of tribe Idaeini in Europe known as detritus feeders);

^(3)^ potential alternative feeding (members of genus *Ergavia* are known as almost exclusively feeding on Polygoniaceae);

^(4)^ potential alternative feeding (members of tribe Lithosiini in Europe known as lichenophagous, genus *Clemensia* known as lichenophagous from North America: Host database).

### Target tree confirming previously known host-plants (larvae without gut content analysis)

Among the 87 larvae successfully identified to genus or species and not subjected to gut content analysis, there are at least six cases where the fogged target trees match previously known host-plant relationships: larvae nr. 63 and 113 (Bombycidae, *Anticla* near *antica*) were knocked down from the trees nr. 14 and 34 (Moraceae); larvae nr. 117 (Geometridae; *Semiothisa gambaria*), 115 (Hesperiidae, *Myscelus*) and 144+126 (Erebidae, *Sosxetra grata*) from the trees nr. 35 and 41 (Meliaceae, *Guarea*), all confirming the relationships as previously recorded by Janzen & Hallwachs [4].

### A powerful tool for future synecological research?

Our pilot study has revealed that (1) molecular identification of fogged, neotropical lepidopteran larvae works successfully in general and even down to species level (if already listed in BOLD), that (2) molecular identification of target trees usually works well at least to genus or family level and (3) molecular gut content analysis based on HTS techniques can be used for confirming or rejecting the feeding on the fogged target tree. With further completion of the DNA reference libraries in the future for (1) (Peruvian Lepidoptera; currently 12,746 sequences, 3532 BINs) and (2) (Peruvian plants) a better taxonomic resolution of identification will be achieved, whilst molecular gut content analysis (3) can be improved by de-contamination and/or isolated storage of the fogged larvae.

With that, the herewith presented approach has the potential for unveiling trophic interactions for primary consumers in tropical regions at a very large scale, which can be performed in a fast and cost-effective way considering the steadily dropping costs for DNA barcoding and HTS. The extremely high diversity of 92 species in 119 larvae in our study shows that canopy fogging and molecular analyses may improve synecological knowledge for a broad spectrum of arthropods. The availability of reliable data on trophic interactions is of great importance for forestry, agriculture, biodiversity and ecological research and–last but not least–for conservation purposes. Increasing such knowledge–particularly in megadiverse ecoregions–is an imperative in a world of unprecedented biodiversity losses. In this context, the proposed molecular approach of investigating host-plant relationships constitutes an important research tool, which fits well in the research plan of the recently launched BIOSCAN phase of the international Barcode of Life program ([40]; see also https://ibol.org).

## Supporting information

S1 Table Morphology-based identification of target trees. Morphology-based identification of target trees to Peruvian vernacular names (mostly provided by the administrator of the Panguana station, Moro Carlos Vásquez Modena) and attempt to assign scientific family / genus / or species names (partly provided by Hamilton Paredes (“HP”), Museum of Natural History, Lima, based on leaf samples). # = fogging sample from target tree without lepidopteran larva; * = no plant tissue available, so far (hence no molecular confirmation possible): ^1^ = same vernacular name with two different molecular identifications.
(PDF)

S2 Table Target trees: Sequencing success and process identification numbers. Data from BOLD, with fragment lengths in basepairs (bp). Sanger sequencing of rbcL, trnL-F and psbA genes, based on leaf (l) and cambium+sapwood (c) samples from the target trees.
(PDF)

S3 Table Molecular identification of target trees. Molecular identification of the target trees after Sanger sequencing (rbcL, trnL-F and psbA genes) of leaf (l) and cambium + sapwood (c) samples. Results from blasting on NCBI, BLAST matches (highest percent identity (‘Max ident’) of all query-subject alignments) usually >99.5%, otherwise indicated. Plant species/genera with blast matches sometimes not mentioned when plants are exclusively distributed on other continents. # = fogging sample from target tree without lepidopteran larva in the sample. Anac. = Anacardiaceae; Anno. = Annonaceae; Cann. = Cannabaceae; Chry. = Chrysobalanaceae; Clus. = Clusiaceae; Euph. = Euphorbiaceae; Faba. = Fabaceae; Malv. = Malvaceae; Malp. = Malpighiaceae; Meli. = Meliaceae; Mora. = Moraceae; Myri. = Myristicaceae; Nyct. = Nyctaginaceae; Rubi. = Rubiaceae; Sapi. = Sapindaceae; Sapo. = Sapotaceae; Viol. = Violaceae. ^1^ = same vernacular name with two different molecular identifications; ^2^ = potential sampling error (tissue sampling from neighboring tree or tube flip in the lab process); ^3^ = exclusively Indo-Pacific; ^4^ = exclusively Old World.
(PDF)
