# Supplementary material for: DNA barcoding of fogged caterpillars in Peru: A novel approach for unveiling host-plant relationships of tropical moths (Insecta, Lepidoptera)
Source: PLoS One. 2020 Jan 30;15(1):e0224188. doi: 10.1371/journal.pone.0224188 (PMC6992181; doi:10.1371/journal.pone.0224188)
Supplement: S1 Table — Morphology-based identification of target trees to Peruvian vernacular names (mostly provided by the administrator of the Panguana station, Moro Carlos Vásquez Modena) and attempt to assign scientific family / genus / or species names (partly provided by Hamilton Paredes (“HP”), Museum of Natural History, Lima, based on leaf samples). # = fogging sample from target tree without lepidopteran larva; * = no plant tissue available, so far (hence no molecular confirmation possible): 1 = same vernacular name with two different molecular identifications. (PDF) [file pone.0224188.s001.pdf]

Supporting information S1 Table. Morphology-based identification of target trees.

Morphology-based identification of target trees to Peruvian vernacular names (mostly provided by the administrator of the Panguana station, "Moro" Carlos Vásquez Módena) and attempt to assign scientific family / genus / or species names (partly provided by Hamilton Paredes ("HP"), Museum of Natural History, Lima, based on leaf samples). # = fogging sample from target tree without lepidopteran larva; * = no plant tissue available, so far (hence no molecular confirmation possible): [1] = same vernacular name with two different molecular identifications.

Target tree nr.	Pre-Identification vernacular name	Pre-Identification tree family	Pre-Identification tree species
1	Mango	Anacardiaceae	*Mangifera indica*
2	Mango	Anacardiaceae	*Mangifera indica*
3	Requia	Meliaceae	*Guarea* species (HP: Annonaceae)
4 #	Ubos	Anacardiaceae	not specified
5	Requia	Meliaceae	*Guarea* species
6 #	Renako	Moraceae	*Ficus* species
7	Ucu muchaca [1]	not specified	not specified
8 #	Peine de mono	Malvaceae	*Apeiba* species
9	Huayaon de mono	Violaceae	*Leonia glycycarpa* (HP)
10	Tortuga	Annonaceae (HP)	not specified
11-1	No vernacular name provided	Cannabaceae	*Celtis schippii* (HP)
11-2	Paucherudo	Nyctaginaceae	*Neea* species (*Guapira*) (HP)
12	Tortuga	Annonaceae (HP)	not specified
13	Tortuga	Annonaceae (HP)	not specified
14	Yanchama	Moraceae	*Poulsenia armata* (HP)
15	Ucu muchaca [1]	not specified	not specified
16	'Kaucho' (cf. Panama rubber tree)	Moraceae	*Castilla*
17	Leche kaspi	Moraceae (HP)	not specified
18	Renako	Moraceae	*Ficus* species
19	Tortuga	Annonaceae (HP)	not specified
20	No vernacular name provided	not specified	not specified
21	Peine de mono	Malvaceae	*Apeiba* species
22	Kaimitio	not specified	not specified
23	No vernacular name provided	not specified	not specified
24	Chimicua	Moraceae	*Perebea*
25	Cumala rosada	Myristicaceae	*Otoba parvifolia*
26 #	Peine de mono	Malvaceae	*Apeiba* species
27	Tortuga	Annonaceae (HP)	not specified
28	Guacamayo caspi	Moraceae	*Ficus* species (HP)

29	Peine de mono	Malvaceae	*Apeiba* species
30 #	Tortuga	Clusiaceae	*Garcinia* (HP)
31-1 #	No vernacular name provided	Clusiaceae	*Garcinia* (HP)
31 -2 #	Tawari	not specified	not specified
32	Espintana blanca	Sapindaceae (HP)	not specified
33	Ucu muchaca [1]	Meliaceae	*Guarea* species (HP)
34	Renako	Moraceae (HP)	not specified
35	Requia blanco	Meliaceae	*Guarea* species (HP)
36	Requia blanco	Meliaceae	*Guarea* species
37	Requia blanco	Meliaceae	*Guarea* species
38	Requia blanco [1]	Meliaceae	*Guarea* species
39 #	Requia blanco [1]	Anacardiaceae	*Tapirira guianensis* (HP)
40	Requia blanco	Meliaceae	*Guarea* species
41	Requia blanco	Meliaceae	*Guarea* species
42	Renako/Chebon	Moraceae	*Ficus* species (HP)
43 #	Requia blanco	Meliaceae	*Guarea* species
44	Requia blanco	Meliaceae	*Guarea* species
45 #	Requia blanco	Meliaceae	*Guarea* species
46 #	Requia blanco	Meliaceae	*Guarea* species
47	Requia blanco	Meliaceae	*Guarea* species
