# Supplementary material for: DNA barcoding of fogged caterpillars in Peru: A novel approach for unveiling host-plant relationships of tropical moths (Insecta, Lepidoptera)
Source: PLoS One. 2020 Jan 30;15(1):e0224188. doi: 10.1371/journal.pone.0224188 (PMC6992181; doi:10.1371/journal.pone.0224188)
Supplement: S2 Table — Data from BOLD, with fragment lengths in basepairs (bp). Sanger sequencing of rbcL, trnL-F and psbA genes, based on leaf (l) and cambium+sapwood (c) samples from the target trees. (PDF) [file pone.0224188.s002.pdf]

Supporting information S2 Table. Target Trees: Sequencing success and process identification numbers. Data from BOLD, with fragment lengths in basepairs (bp). Sanger sequencing of rbcL, trnL-F and psbA genes, based on leaf (l) and cambium+sapwood (c) samples from the target trees.

Target tree nr.	Process ID rbcL (CCDB), from leaves (l) & cambium (c)	Fragment length	Process ID trnL-F (CCDB), from leaves (l) & cambium (c)	Fragment length	Process ID rbcL (AIM), from leaves	Fragment length	Process ID psbA (AIM), from leaves	Fragment length	Sequence information total
1	PANFT001-18 (l)<br>PANFT036-18 (c)	553 bp<br>553 bp	PANFT001-18 (l)<br>PANFT036-18 (c)	493 bp<br>493 bp	PANFT096-18	617 bp	failure	0 bp	1110 bp
2	PANFT037-18 (c)	553 bp	PANFT002-18 (l)<br>PANFT037-18 (c)	493 bp<br>493 bp	PANFT097-18	684 bp	PANFT117-18	549 bp	1726 bp
3	PANFT003-18 (l)<br>PANFT007-18 (l)<br>PANFT038-18 (c)	553 bp<br>522 bp<br>553 bp	PANFT038-18 (c)	537 bp	failure	0 bp	failure	0 bp	1090 bp
4 #	PANFT004-18 (l)<br>PANFT039-18 (c)	553 bp<br>553 bp	PANFT039-18 (c)	557 bp	failure	0 bp	failure	0 bp	1110 bp
5	PANFT040-18 (c)	553 bp	PANFT040-18 (c)	557 bp	failure	0 bp	failure	0 bp	1110 bp
6 #	PANFT013-18 (l)<br>PANFT041-18 (c)	553 bp<br>553 bp	PANFT013-18 (l)<br>PANFT041-18 (c)	541 bp<br>541 bp	failure	0 bp	failure	0 bp	1094 bp
7	PANFT014-18 (l)<br>PANFT042-18 (c)	553 bp<br>553 bp	PANFT014-18 (l)<br>PANFT042-18 (c)	535 bp<br>557 bp	PANFT098-18	696 bp	failure	0 bp	1231 bp
8 #	PANFT043-18 (c)	553 bp	PANFT043-18 (c)	587 bp	no leaf provided	0 bp	no leaf provided	0 bp	1110 bp
9	PANFT015-18 (l)<br>PANFT044-18 (c)	553 bp<br>553 bp	PANFT015-18 (l)<br>PANFT044-18 (c)	561 bp<br>305 bp	PANFT099-18	682 bp	PANFT118-18	470 bp	1713 bp
10	PANFT016-18 (l)<br>PANFT045-18 (c)	553 bp<br>553 bp	PANFT016-18 (l)<br>PANFT045-18 (c)	536 bp<br>536 bp	PANFT100-18	694 bp	PANFT119-18	545 bp	1775 bp
11-1	PANFT017-18 (l)<br>PANFT046-18 (c)	553 bp<br>553 bp	PANFT017-18 (l)<br>PANFT046-18 (c)	553 bp<br>553 bp	PANFT101-18	687 bp	PANFT120-18	598 bp	1838 bp
11-2	PANFT018-18 (l)	553 bp	PANFT018-18 (l)	514 bp	PANFT102-18	637 bp	PANFT121-18	563 bp	1714 bp
12	PANFT019-18 (l)<br>PANFT047-18 (c)	553 bp<br>553 bp	PANFT019-18 (l)<br>PANFT047-18 (c)	536 bp<br>574 bp	PANFT103-18	690 bp	PANFT122-18	526 bp	1790 bp
13	PANFT019-18 (l)<br>PANFT048-18 (c)	failure<br>553 bp	PANFT019-18 (l)<br>PANFT048-18 (c)	failure<br>536 bp	PANFT104-18	628 bp	PANFT123-18	550 bp	1714 bp
14	PANFT021-18 (l)<br>PANFT049-18 (c)	553 bp<br>553 bp	PANFT021-18 (l)<br>PANFT049-18 (c)	535 bp<br>535 bp	PANFT105-18	663 bp	failure	0 bp	1198 bp
15	PANFT022-18 (l)<br>PANFT050-18 (c)	553 bp<br>553 bp	PANFT022-18 (l)<br>PANFT050-18 (c)	223 bp<br>failure	PANFT106-18	691 bp	failure	0 bp	914 bp
16	PANFT051-18 (c)	553 bp	PANFT051-18 (c)	536 bp	no leaf provided	0 bp	no leaf provided	0 bp	1089 bp
17	PANFT023-18 (l)<br>PANFT052-18 (c)	553 bp<br>553 bp	PANFT023-18 (l)<br>PANFT052-18 (c)	539 bp<br>539 bp	PANFT107-18	688 bp	PANFT124-18	490 bp	1717 bp
18	PANFT053-18 (c)	553 bp	PANFT053-18 (c)	541 bp	Failure	0 bp	Failure	0 bp	1094 bp
19	PANFT024-18 (l)<br>PANFT054-18 (c)	553 bp<br>553 bp	PANFT024-18 (l)<br>PANFT054-18 (c)	536 bp<br>536 bp	PANFT108-18	627 bp	PANFT125-18	562 bp	1725 bp
20	PANFT055-18 (c)	553 bp	PANFT055-18 (c)	443 bp	no leaf provided	0 bp	no leaf provided	0 bp	996 bp
21	PANFT056-18 (c)	553 bp	PANFT056-18 (c)	526 bp	no leaf provided	0 bp	no leaf provided	0 bp	1079 bp
22	PANFT057-18 (c)	553 bp	PANFT057-18 (c)	537 bp	no leaf provided	0 bp	no leaf provided	0 bp	1090 bp
23	no tissue provided	0 bp	no tissue provided	0 bp	no leaf provided	0 bp	no leaf provided	0 bp	0 bp
24	PANFT058-18 (c)	553 bp	PANFT058-18 (c)	543 bp	no leaf provided	0 bp	no leaf provided	0 bp	1096 bp
25	PANFT059-18 (c)	553 bp	PANFT059-18 (c)	536 bp	no leaf provided	0 bp	no leaf provided	0 bp	1089 bp
26 #	PANFT060-18 (c)	553 bp	PANFT060-18 (c)	587 bp	no leaf provided	0 bp	no leaf provided	0 bp	1140 bp
27	PANFT025-18 (l)<br>PANFT061-18 (c)	553 bp<br>553 bp	PANFT025-18 (l)<br>PANFT061-18 (c)	536 bp<br>536 bp	PANFT109-18	691 bp	PANFT126-18	547 bp	1774 bp
28	PANFT026-18 (l)<br>PANFT062-18 (c)	553 bp<br>553 bp	PANFT026-18 (l)<br>PANFT062-18 (c)	541 bp<br>535 bp	PANFT110-18	687 bp	failure	0 bp	1228 bp
29	PANFT063-18 (c)	553 bp	PANFT063-18 (c)	587 bp	no leaf provided	0 bp	no leaf provided	0 bp	1140 bp
30 #	PANFT027-18 (l)<br>PANFT064-18 (c)	358 bp<br>553 bp	PANFT064-18 (c)	138 bp	PANFT111-18	674 bp	failure	0 bp	812 bp

31-1 #	PANFT028-18 (l)	553 bp	failure	0 bp	PANFT112-18	686 bp	failure	0 bp	686 bp
31-2 #	PANFT029-18 (l)<br>PANFT065-18 (c)	553 bp<br>553 bp	PANFT029-18 (l)<br>PANFT065-18 (c)	543 bp<br>561 bp	failure	0 bp	failure	0 bp	1114 bp
32	PANFT030-18 (l)<br>PANFT066-18 (c)	553 bp<br>553 bp	PANFT030-18 (l)<br>PANFT066-18 (c)	573 bp<br>536 bp	PANFT113-18	668 bp	PANFT127-18	518 bp	1759 bp
33	PANFT031-18 (l)<br>PANFT067-18 (c)	553 bp<br>553 bp	PANFT031-18 (l)<br>PANFT067-18 (c)	557 bp<br>557 bp	failure	0 bp	failure	0 bp	1110 bp
34	PANFT032-18 (l)<br>PANFT068-18 (c)	553 bp<br>553 bp	PANFT032-18 (l)<br>PANFT068-18 (c)	541 bp<br>541 bp	failure	0 bp	failure	0 bp	1094 bp
35	PANFT033-18 (l)<br>PANFT069-18 (c)	553 bp<br>553 bp	PANFT033-18 (l)<br>PANFT069-18 (c)	557 bp<br>557 bp	PANFT114-18	641 bp	PANFT128-18	514 bp	1712 bp
36	PANFT070-18 (c)	553 bp	PANFT070-18 (c)	557 bp	failure	0 bp	failure	0 bp	1110 bp
37	PANFT071-18 (c)	553 bp	PANFT071-18 (c)	557 bp	failure	0 bp	failure	0 bp	1110 bp
38	PANFT072-18 (c)	553 bp	PANFT072-18 (c)	366 bp	failure	0 bp	failure	0 bp	919 bp
39 #	PANFT034-18 (l)<br>PANFT073-18 (c)	553 bp<br>553 bp	PANFT034-18 (l)<br>PANFT073-18 (c)	566 bp<br>557 bp	PANFT115-18	509 bp	failure	0 bp	1119 bp
40	PANFT074-18 (c)	553 bp	PANFT074-18 (c)	557 bp	failure	0 bp	failure	0 bp	1110 bp
41	PANFT075-18 (c)	553 bp	PANFT075-18 (c)	557 bp	failure	0 bp	failure	0 bp	1110 bp
42	PANFT035-18 (l)<br>PANFT076-18 (c)	553 bp<br>553 bp	PANFT035-18 (l)<br>PANFT076-18 (c)	541 bp<br>541 bp	PANFT116-18	685 bp	failure	0 bp	1226 bp
43 #	PANFT077-18 (c)	553 bp	PANFT077-18 (c)	557 bp	failure	0 bp	failure	0 bp	1110 bp
44	PANFT078-18 (c)	553 bp	PANFT078-18 (c)	557 bp	failure	0 bp	failure	0 bp	1110 bp
45 #	PANFT079-18 (c)	553 bp	PANFT079-18 (c)	557 bp	failure	0 bp	failure	0 bp	1110 bp
46 #	PANFT080-18 (c)	553 bp	PANFT080-18 (c)	557 bp	failure	0 bp	failure	0 bp	1110 bp
47	PANFT081-18 (c)	553 bp	PANFT081-18 (c)	557 bp	failure	0 bp	failure	0 bp	1110 bp

6
