# Supplementary material for: DNA barcoding of fogged caterpillars in Peru: A novel approach for unveiling host-plant relationships of tropical moths (Insecta, Lepidoptera)
Source: PLoS One. 2020 Jan 30;15(1):e0224188. doi: 10.1371/journal.pone.0224188 (PMC6992181; doi:10.1371/journal.pone.0224188)
Supplement: S3 Table — Molecular identification of the target trees after Sanger sequencing (rbcL, trnL-F and psbA genes) of leaf (l) and cambium + sapwood (c) samples. Results from blasting on NCBI, BLAST matches (highest percent identity (‘Max ident’) of all query-subject alignments) usually >99.5%, otherwise indicated. Plant species/genera with blast matches sometimes not mentioned when plants are exclusively distributed on other continents. # = fogging sample from target tree without lepidopteran larva in the sample. Anac. = Anacardiaceae; Anno. = Annonaceae; Cann. = Cannabaceae; Chry. = Chrysobalanaceae; Clus. = Clusiaceae; Euph. = Euphorbiaceae; Faba. = Fabaceae; Malv. = Malvaceae; Malp. = Malpighiaceae; Meli. = Meliaceae; Mora. = Moraceae; Myri. = Myristicaceae; Nyct. = Nyctaginaceae; Rubi. = Rubiaceae; Sapi. = Sapindaceae; Sapo. = Sapotaceae; Viol. = Violaceae. 1 = same vernacular name with two different molecular identifications; 2 = potential sampling error (tissue sampling from neighboring tree or tube flip in the lab process); 3 = exclusively Indo-Pacific; 4 = exclusively Old World. (PDF) [file pone.0224188.s003.pdf]

Supporting information S3 Table. Molecular identification of target trees. Molecular identification of the target trees after Sanger sequencing (rbcL, trnL-F and psbA genes) of leaf (l) and cambium+sapwood (c) samples. Results from blasting on NCBI, BLAST matches (highest percent identity ('Max ident') of all query-subject alignments) usually >99.5%, otherwise indicated. Plant species/genera with blast matches sometimes not mentioned when plants are exclusively distributed on other continents. # = fogging sample from target tree without lepidopteran larva in the sample. [1] = same vernacular name with two different molecular identifications; [2] = potential sampling error (tissue sampling from neighboring tree or tube flip in the lab process); [3] = exclusively Indo-Pacific; [4] = exclusively Old World.

Target tree nr.	Molecular identification rbcL (CCDB), from leaves (l) & cambium (c)	Molecular identification trnL-F (CCDB), from leaves (l) & cambium (c)	Molecular identification rbcL (AIM), from leaves	Molecular identification psbA (AIM), from leaves	Plant family
1	*Mangifera indica* (l+c)	*Mangifera indica* (l+c)	*Mangifera indica*	failure	Anacardiaceae
2	*Mangifera indica* (l+c)	*Mangifera indica* (l+c)	*Mangifera indica*	*Mangifera indica*	Anacardiaceae
3	*Mangifera indica, Spondias* (l [2]); *Cabralea, Dysoxylum* [3] etc. (c)	*Dysoxylum* [3], *Chisocheton* [3], *Guarea* (c)	failure	failure	Anacardiaceae (CCDB/rbcL: l), Meliaceae (CCDB/rbcL+trnL-F: c)
4#	*Mangifera indica* (l) *Spondias* (99.0%) (c)	*Dysoxylum* [3], *Chisocheton* [3], *Guarea* (c [2])	failure	failure	Anacardiaceae (CCDB/rbcL: l+c), Meliaceae (CCDB/trnL-F: c)
5	*Cabralea, Dysoxylum* etc. (c)	*Dysoxylum* [3], *Chisocheton* [3], *Guarea* (c)	failure	failure	Meliaceae
6#	*Ficus* spec. (l+c)	*Ficus carica / microcarpa* (99.6%). (l+c)	failure	failure	Moraceae
7 [1]	*Ochroma, Bombax* [4], *Heritiera,* etc. (l [1] [2]), *Trichilia* (c [2])	*Quararibea* (98%) (l [2]), *Trichilia* (c [2])	*Ochroma pyramidale* (99.7%), *Bombax* [4] (99.6%)	failure	Malvaceae (CCDB/rbcL+trnL-F: l; AIM/rbcL: l), Meliaceae (CCDB/rbcL+trnL-F: c)
8#	*Apeiba, Goethalsia, Theo-broma cacao* (c)	*Corchorus*[4] (96.1%) (c)	no leaf provided	no leaf provided	Malvaceae
9	*Leonia glycycarpa, Amphirrhox* (l+c)	*Leonia glycycarpa* (99.7% / 98.9%) (l+c)	*Leonia glycycarpa*	*Leonia glycycarpa*	Violaceae
10	*Oxandra euneura / polyantha* (l+c)	*Oxandra polyantha* (l+c)	*Oxandra euneura / polyantha*	*Oxandra lanceolata*	Annonaceae
11-1	*Celtis schippii* (l+c)	Cannabaceae (94.8%) (l+c)	*Celtis schippii*	*Celtis schippii*	Cannabaceae
11-2	*Neea, Guapira, Pisonia,* etc. (l)	*Neea floribunda* (97.5%) (l)	*Neea*	failure	Nyctaginaceae

12	*Oxandra euneura / polyantha* (l) – *Conceveiba, Aparisthium* (c [2])	*Oxandra polyantha* (l) – *Conceveiba guianensis* (c [2])	*Oxandra euneura / polyantha*	*Oxandra lanceolata*	Annonaceae (CCDB/rbcL+trnL-F: l; AIM/rbcL+psbA: l), Euphorbiaceae (CCDB/rbcL+trnL-F: c)
13	*Oxandra euneura / polyantha* (c)	*Oxandra polyantha* (c)	*Oxandra euneura / polyantha*	*Oxandra lanceolata*	Annonaceae
14	*Pseudolmedia, Castilla, Naucleopsis* (l+c)	*Naucleopsis guianensis*, *Helicostylis tomentosa* (l+c)	*Perebea guianensis* (99.6%)	failure	Moraceae
15 [1]	*Hirtella* (6 species 100%) (l+c) [1 3]	*Hirtella*	*Hirtella*	failure	Chrysobalanaceae
16	*Castilla* (100%), *Pseudolmedia* (99.8%), *Naucleopsis* (99.6%) (c)	*Castilla elastica* (100%) (c)	no leaf provided	no leaf provided	Moraceae
17	*Clarisia biflora* (l+c)	*Batocarpus* (99.3%) (c)	*Clarisia racemosa*	*Clarisia biflora*	Moraceae
18	*Ficus* > 30 species (all 100%) (c)	*Ficus carica, F. religiosa, F. sarmentosa* [3], *F. microcarpa* (all 99.6%) (c)	failure	failure	Moraceae
19	*Oxandra euneura / polyantha* (l+c)	*Oxandra polyantha* (l+c)	*Oxandra asbeckii / euneura / polyantha* (99.4%)	*Oxandra lanceolata*	Annonaceae
20	*Neea, Guapira, Pisonia,* etc. (c)	*Neea floribunda* (100%) (c)	no leaf provided	no leaf provided	Nyctaginaceae
21	*Annona rugulosa* (99.8%), *A. coriacea* (99.6%) (c)	*Annona amazonica, A. symphyocarpa* (both 99.8%) (c)	no leaf provided	no leaf provided	Annonaceae
22	*Byrsonima coccolobifolia* (c)	*Byrsonima coccolobifolia* (c)	no leaf provided	no leaf provided	Malpighiaceae
23	no tissue provided	no tissue provided	no leaf provided	no leaf provided	no tissue provided
24	*Pouteria, Chrysophyllum* (c)	*Pouteria, Chrysophyllum* (c)	no leaf provided	no leaf provided	Sapotaceae
25	*Myristica, Virola, Knema, Horsfieldia* (c)	*Myristica elliptica* (99.6%) (c)	no leaf provided	no leaf provided	Myristicaceae
26#	*Theobroma* (99.5%), *Herrania* (99.3%) (c)	*Corchorus* (96.0%) (c)	no leaf provided	no leaf provided	Malvaceae
27	*Oxandra euneura / polyantha* (l+c)	*Oxandra polyantha* (l+c)	*Oxandra euneura / polyantha*	*Oxandra lanceolata*	Annonaceae
28	*Ficus* (Mora.) (l) – *Alseis* (99.8%), *Simira, Pentagonia* (99.6%) (all Rubi.) (c) [2]	*Ficus* (Mora.) (l) – *Simira* (100%) (Rubi.) (c) [2]	*Ficus* spec. (Mora.)	failure	Moraceae (CCDB/rbcL+trnL-F: l; AIM/rbcL: l), Rubiaceae (CCDB/rbcL+trnL-F: c)
29	*Theobroma* (99.5%), *Herrania* (99.3%) (c)	*Corchorus* (96.1%) (c)	no leaf provided	no leaf provided	Malvaceae
30#	*Garcinia macrophylla* (99.8%) (l+c)	*Garcinia mangostana* (100%) (c)	*Garcinia* (Clus.)	failure	Clusiaceae
31-1#	*Garcinia macrophylla* (99.6%) (l+c)	failure	*Garcinia*	failure	Clusiaceae
31-2#	*Manilkara, Chrysophyllum, Pouteria* (l) – *Andira inermis* (c) [2 3]	*Manilkara, Chrysophyllum, Pouteria* etc. (l) - *Andira inermis* (c)[2 3]	failure	failure	Sapotaceae (CCDB/rbcL+trnL-F: l); Fabaceae (CCDB/rbcL+trnL-F: c)
32	*Paullinia cururu / bracteosa* (100%) (Sapi.) (l) – *Oxandra sphaerocarpa, O. macrophylla* (Anno.) (c) [2]	*Paullinia elegans* (100%) – *Oxandra macrophylla* (100%), *O. riedeliana* (99.8%) (Anno.) (c) [2]	*Paullinia globosa* (99.7%) (Sapi.)	*Paullinia cururu* (99%) (Sapi.)	Sapindaceae (CCDB/rbcL+trnL-F: l; AIM/rbcL+psbA: l); Annonaceae (CCDB/rbcL+trnL-F: c)

33 [1]	*Trichilia* (l+c)	*Trichilia* (l+c)	failure	failure	Meliaceae
34	*Ficus* > 30 species (all 100%) (l+c)	*Ficus carica, F. religiosa, F. sarmentosa* [3]*, F. microcarpa* (all 99.6%) (c)	failure	failure	Moraceae
35	*Guarea guidonia, G. macrophylla* (both 99.8%) (l+c)	*Guarea guidonia* (100%) (l+c)	*Tapirira mexicana* (99.5%)[2]	*Guarea guidonia* (98%)	Meliaceae (CCDB/rbcL+trnL-F: l+c; AIM/psbA: l); Anacardiaceae (AIM/rbcL: l)
36	*Guarea guidonia, G. macrophylla* (both 99.8%) (c)	*Guarea guidonia* (100%) (c)	failure	failure	Meliaceae
37	*Guarea guidonia, G. macrophylla* (both 99.8%) (c)	*Guarea guidonia* (100%) (c)	failure	failure	Meliaceae
38 [1]	*Erythrina* (c) [1 2]	*Erythrina speciosa* (c) [1 2]	failure	failure	Fabaceae
39# [1]	*Tapirira guianensis* (l) – *Guarea guidonia, G. macrophylla* (both 99.8%) (c) [1 2]	*Tapirira guianensis* (100%) (l) – *Guarea guidonia* (Meli.) (c) [1 2]	*Tapirira* (98.5%)	failure	Anacardiaceae (CCDB/rbcL+trnL-F: l; AIM/rbcL: l); Meliaceae (CCDB/rbcL+trnL-F: c)
40	*Guarea guidonia, G. macrophylla* (both 99.8%) (c)	*Guarea guidonia* (100%) (c)	failure	failure	Meliaceae
41	*Guarea guidonia, G. macrophylla* (both 99.8%) (c)	*Guarea guidonia* (100%) (c)	failure	failure	Meliaceae
42	*Ficus* > 30 species (l+c)	*Ficus carica, F. religiosa, F. sarmentosa* [3]*, F. microcarpa* (all 99.6%) (c)	*Ficus* spec.	failure	Moraceae
43#	*Guarea guidonia, G. macrophylla* (both 99.8%) (c)	*Guarea guidonia* (100%) (c)	failure	failure	Meliaceae
44	*Guarea guidonia, G. macrophylla* (both 99.8%) (c)	*Guarea guidonia* (100%) (c)	failure	failure	Meliaceae
45#	*Guarea guidonia, G. macrophylla* (both 99.8%) (c)	*Guarea guidonia* (100%) (c)	failure	failure	Meliaceae
46#	*Guarea guidonia, G. macrophylla* (both 99.8%) (c)	*Guarea guidonia* (100%) (c)	failure	failure	Meliaceae
47	*Guarea guidonia, G. macrophylla* (both 99.8%) (c)	*Guarea guidonia* (100%) (c)	failure	failure	Meliaceae
